# Effects of a blended design of closed-book and open-book examinations on dental students’ anxiety and performance

**DOI:** 10.1186/s12909-023-04014-9

**Published:** 2023-01-13

**Authors:** Sumin Hong, Bokyung Go, Jaehee Rho, Soyoun An, Cheolil Lim, Deog-Gyu Seo, Jungjoon Ihm

**Affiliations:** 1 Department of Education, Seoul National University, Seoul, Korea; 2 Department of Education, College of Educational Science, Yonsei University, Seoul, Korea; 3 Department of Pediatric Dentistry &, College of Dentistry, Wonkwang Bone Regeneration Research Institute, Wonkwang University, Iksan-Si, Korea; 4 Dental Research Institute, School of Dentistry, Seoul National University, Gwanak-Ro 1, Gwanak-Gu, Seoul, Republic of Korea; 5 Department of Conservative Dentistry, School of Dentistry, Seoul National University, 101 Daehakno, Jongno-Gu, Seoul, Republic of Korea; 6 Department of Dental Education, School of Dentistry, Seoul National University, Seoul, Korea

**Keywords:** Blended assessment, Open-book exams, Closed-book exams, Test anxiety, Academic performance

## Abstract

**Background:**

While closed-book examinations (CBEs) have traditionally been implemented in dental education, open-book examinations (OBEs) are being introduced for the purpose of acquiring higher levels of knowledge and promoting long-term memory. This study examines whether it is effective to use a blended assessment of CBEs and OBEs for dental students to reduce test anxiety and enhance academic performance.

**Methods:**

Using a quasi-experimental research method, a blended assessment that combined CBEs in class and OBEs online was designed for a dental course. In 2020, when the pandemic was at its peak, student assessment was ineffective, and the 2020 cohort was omitted for our study; instead, two cohorts of predoctoral dental students (*N* = 178) enrolled in Restorative Dentistry in the spring semesters of 2019 and 2021 were included in the study. These students were informed about the experimental design, and they provided written consent for data collection, thereby voluntarily participating in the survey. Their self-perceived responses to open-ended survey questions on assessment methods were qualitatively analyzed.

**Results:**

There was no significant difference in test anxiety between the CBEs and OBEs at the *p*-values of 0.001 in all items. Traditional and blended assessment showed a similar trend of lower scores in midterm exams compared to higher scores in final exams, thus discriminating against students’ performances. In particular, a low-achieving group was better predicted by a blended assessment. An analysis of the students’ self-perceived responses produced highly topical themes, including exam burden, learning effects, and fairness issues.

**Conclusions:**

This study confirmed the feasibility of blended assessment that can be implemented in online and in-person educational environments. Moreover, it can be used as the groundwork to develop new models of assessment in dental education.

## Background

Closed-book examinations (CBEs) are more preferred than open-book examinations (OBEs), and CBEs are considered as the common assessment in medical and dental schools [1]. Due to the challenges posed by the COVID-19 pandemic to in-person instruction and assessment in the online environment, medical educators have begun to implement OBEs as an alternative form of assessment in their online courses [2]. As views regarding what is required to be a competent healthcare professional are changing, educators must understand the pros and cons of OBEs and CBEs.

The concept of OBEs is not entirely new, and it is related to the higher-order thinking of taxonomy, in which Bloom et al. in 1935 distinguished the levels of cognitive thinking domains. OBEs do not stop at simply memorizing knowledge for learners, but they also evaluate whether the acquired knowledge can be used at a higher cognitive level [3]. Beyond measuring the memorization of knowledge, OBEs can evaluate higher-order cognitive abilities such as application, analysis, and evaluation [4, 5]. This type of exam stimulates students’ long-term memory and helps them improve the effectiveness of their learning by developing metacognition to enable the extension of memory for longer periods [6].

However, proponents of CBEs assert that students tend to invest more time and mental efforts in preparing for closed-book exams, stressing that medical students’ expert performance is closely linked to well-organized subject knowledge [2]. This argument gives rise to question of whether open-book items can accurately measure the cognitive level of students [7]. Another problem is that when students are assessed through OBEs, the lower tension is likely to result in lower learning duration and learning volume [7]. On the other hand, opponents of CBEs argue that it primarily aims to store information for quick retrieval and that rote memorization leads to superficial learning [8]. This type of learning ends up cramming that results in dumping information immediately after tests [2, 7].

It is still an open question which approach is more beneficial to learning, as CBEs and OBEs have had mixed effects, exhibiting both benefits and costs [9–11]. Previous studies have found that dental students who take OBEs tend to become more responsible and self-directed learners [12]. A survey of student perceptions recently conducted regarding OBEs in a UK dental school showed that dental students with a strong preference for OBEs showed greater effectiveness in their learning than those who preferred CBEs [7]. More importantly, since text anxiety may be associated with the experience of negative emotions during exams, it is important to note that OBEs can lower learners’ anxiety during assessments, thus enabling higher performance [3, 6, 7, 13].

Specifically, unlike previous studies, which have simply proposed one-way OBEs, this study compared a blended CB-OB exam format with traditional CB formats and examined the applicability of this approach in online settings. To the best of our knowledge, there is a lack of studies with experimental designs showing the effects of a blended approach of CB and OB tests on dental students’ outcomes. As competency-based dental education assesses competencies other than the maintenance of dental knowledge, more attention must be paid to blended assessments in education for the health professions. This approach assessed here consists of, first, a CBE that evaluates concepts that students should know even without resources being available, followed by a second half, open-book assessment that capitalizes on real problems that students will be expected to solve with available resources. Specifically, the CBE format used true or false, multiple-choice, and short-answer questions, but essay questions were designed for the OBE format.

This study investigated whether a blended approach involving CBEs in class and OBEs online is more effective than traditional assessments in dental education. In 2020, when the pandemic was at its peak, every dental class was conducted online and instructors were unprepared to assess students’ learning online; thus, the 2020 cohort for the same course was omitted for our study. Instead, we compared different cohorts taking the same course led by the same instructors in the 2019 pre-pandemic class using CBE and 2021 post-pandemic class using blended CBE and OBE.

To confirm the feasibility of a blended approach, this study examined potential differences in students’ test anxiety level between midterm in-person CBEs and final online OBEs in a dental course in 2021 and then compared the distribution of their academic performance between the blended assessment in 2021 and the traditional one in 2019 for the same course. In particular, 2021’s final OBEs were conducted online due to the COVID-19 pandemic. Students’ self-perceived responses to open-ended questions concerning blended assessment were qualitatively analyzed.

## Methods and materials

### Research design

We examined the effects of a blended assessment on dental students’ anxiety and performance using a quasi-experimental research methodology. The students were fully informed of our experimental design by means of the course syllabus before the class began and provided written informed consent to the collection of data and voluntarily participated in the survey. The study was approved by the Institutional Review Board of the School of Dentistry, Wonkwang University, Republic of Korea (Institutional Review Board No. WKIRB-202009-BM-062), and it adhered to university policy on research with human participants.

### Procedure and setting

The students who took the course in Restorative Dentistry offered by the university in 2019 took the midterm and final exams as CBEs, while in the same course administered a blended CBE and OBE assessment in 2021. This course generally covers the theoretical basis and formulas of direct and indirect methods of aesthetically recovering damaged teeth. The midterm CBEs in 2021 were administered in a face-to-face setting with students physically attending class just like it was before the COVID-19 pandemic, time was limited to a duration of one hour, and the questions included true or false questions, factual knowledge multiple-choice questions, and short-answer questions.

For the 2021 final exams, the students took OBEs with textbooks available in an online setting. The OBEs consisted of three questions that aimed at assessing the students’ problem-solving skills. For example, the students were asked to check the patient’s condition and work out the patient’s treatment plans. Several measures were put in place to secure the validity of the OBEs and prevent exam irregularities. During the one-hour online open-book test, the students were required to keep their cameras on to show their faces on Zoom Meetings, a cloud-based video conferencing platform. To enable impartial evaluation, plagiarism was detected using the Turnitin program, a web-based plagiarism prevention service. The 2021 scores of the midterm CBEs and final OBEs were then examined and compared with the 2019 scores of full CBEs under the same course.

### Data analysis

In this study, to prove the feasibility of the assessment methods, the test anxiety level and exam scores were compared on average within the same group, and therefore, the researchers conducted the paired sample t-test in 2019 and 2021, respectively. The study also attempted to confirm the relationship between the test formats and the learners’ cumulative grade point average (GPA) by dividing the students’ GPAs into two groups (i.e., high achievers and low achievers) based on a median split. A linear regression was calculated to predict the exam scores in 2019 and 2021, and data analysis was conducted using SPSS version 23 (SPSS Inc., Chicago, IL, USA).

To examine students’ self-perceived responses to the blended CB-OB exams in 2021, the data collected through the open-ended survey questions were qualitatively analyzed in three stages: transcription, coding, and theme identification. The two researchers, who were now familiar with the students’ overall perceptions, performed open coding by making lists of responses and categorizing them, developing themes to encompass its meaning. We reviewed the research results using methodological triangulation to increase the validity and reliability of qualitative research, taking into account multiple perspectives, frequencies, and contexts of the results [14].

### Participants

A total of 178 predoctoral dental students who enrolled in Restorative Dentistry during the spring semester of 2019 and 2021 signed an informed consent to the collection of their records. The population was the total group of students enrolled in the class, and those who agreed to the study and had no history of mental illness were sampled. In the performance of a paired t-test, the sample size must be 27 or larger to identify a medium effect size and to have a power of 0.8 or higher. This study had a sample of 89 participants, indicating a sufficient level of power. The 2021’s sample contained 56 (62.9%) male and 33 (37.1%) female students, and the 2019’s sample contained 50 (56.2%) male and 39 (43.8%) female students.

### Measures

#### Test anxiety

To measure test anxiety, Benson and El-Zahhar’s Revised Test Anxiety Scale (RTA) was used [15]. The RTA consists of 4 factors and 20 questions, including 5 questions on tension, 6 on the worry, 5 on the physical symptom, and 4 on the thinking unrelated to the test. The learners’ answers to the test anxiety questions were based on a four-point scale, ranging from rarely feeling (1) to always feeling (4). The reliability for the test anxiety portion was assessed with Cronbach’s alpha value of 0.932 at the midterm exam and 0.954 at the final exam. To verify the validity of the questionnaire, a Pearson correlation coefficient was identified for each item score and the total score, and the coefficients were 0.536 to 0.865 in the midterm exam and 0.613 to 0.833 in final exam. All items were significantly correlated with the total score, indicating that all items were valid. Table 1 shows the descriptive statistics from the 2021 sampling.

**Table 1:** Dental students’ test anxiety comparison between in-person CBEs and online OBEs

	Test anxiety items	CBEs (*n* = 89)		OBEs (*n* = 87)	
		M	SD	M	SD
1	Thinking about my grade in a course interferes with my work on tests	2.61	0.94	2.52	0.93
2	I seem to defeat myself while taking important tests	2.18	0.96	2.17	0.81
3	During tests I find myself thinking about the consequences of failing	2.36	0.92	2.33	0.95
4	I start feeling very uneasy just before getting a test paper back	2.19	0.95	2.15	0.97
5	During tests I feel very tense	2.63	0.93	2.47	0.95
6	I worry a great deal before taking an important exam	2.71	0.94	2.80	0.91
7	During tests I find myself thinking of things unrelated to the material being tested	1.93	0.86	1.93	0.86
8	While taking tests, I find myself thinking how much brighter the other people are	2.27	1.13	2.29	1.02
9	I think about current events during a test	1.74	0.81	1.86	0.88
10	I get a headache during an important test	1.89	0.91	1.94	0.98
11	While taking a test, I often think about how difficult it is	2.49	0.84	2.41	0.83
12	I am anxious about tests	2.52	0.92	2.55	0.89
13	While taking tests I sometimes think about being somewhere else	1.61	0.87	1.67	0.88
14	During tests I find I am distracted by thoughts of upcoming events	1.62	0.80	1.76	0.90
15	My mouth feels dry during a test	1.98	1.04	1.93	1.03
16	I sometimes find myself trembling before or during tests	1.92	0.96	1.92	1.00
17	While taking a test my muscles are very tight	1.79	0.89	1.91	1.00
18	I have difficulty breathing while taking a test	1.25	0.63	1.40	0.75
19	During the test I think about how I should have prepared for the test	2.30	0.91	2.44	0.91
20	I worry before the test because I do not know what to expect	2.18	0.92	2.22	0.97

Data are shown as mean values (M) and standard deviations (SD)

#### Perception survey on a blended assessment

To confirm the self-perception of the blended assessment, open-ended survey questions were developed and validated by four experts, including two clinical professors and two professors majoring in dental education. The survey featured open-ended questions concerning exam preparation, the perceived effect of the format on student learning, and students’ exam experiences. The students provided their perceptions in narrative form.

## Results

### Test anxiety comparison

No statistically different mean difference in the test anxiety level of a blended assessment was found between the in-person CBEs and the online OBEs. The mean anxiety level for the midterm CBEs was 2.108, and the level for the final OBEs was 2.134, an increase of 0.026 points. There was no significant difference in the *p*-values of 0.001 in all items.

### Academic achievement comparison

#### Comparison between midterm CBEs and final CBEs in 2019 (before COVID-19)

When comparing the midterm with the final exam scores for Restorative Dentistry in 2019, the t-test showed a significant difference (t (88) =  − 16.02, *p* < 0.001). The mean score of the midterm CBEs was 75.79 (SD = 7.96) and that of the final CBEs was 92.72 (SD = 9.48).

#### Comparison between midterm CBEs and final OBEs in 2021 (under COVID-19)

When comparing the midterm scores with the final exam scores for Restorative Dentistry in 2021, the t-test showed a significant difference (t (88) =  − 8.01, *p* < 0.001). The mean score of the in-person midterm CBEs was 79.87 (SD = 4.92) and that of the online final OBEs was 87.88 (SD = 8.13).

#### Total score comparison between CBEs in 2019 and blended CBEs-OBEs in 2021

**Fig. 1:**
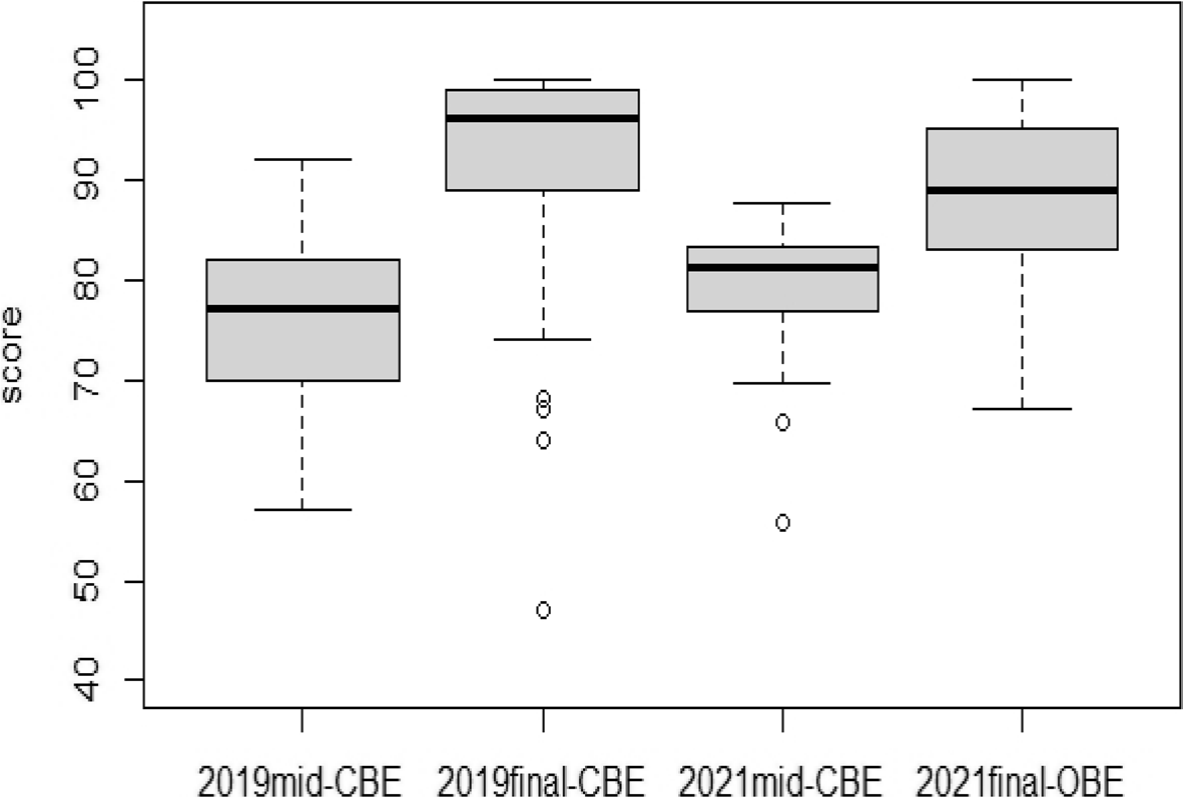
Box plot of 2019 CBE and 2021 blended exam scores for the Restorative Dentistry course (2019 *n* = 89, 2021 *n* = 89)

To compare the academic achievement of the same course in 2019 and 2021, the researchers drew a boxplot in Fig. 1. Both years showed a similar trend of lower scores in midterm exams compared to the high scores exhibited in the final exams. Regarding the midterm scores, the standard deviation of 2021 was smaller than that of 2019. The online OB final scores in 2021 were less skewed than the in-person CB final scores in 2019.

##### Prediction of high or low achiever’s performances on blended test scores

Table 2 shows that 2019’s traditional CB scores and 2021’s blended CB-OB scores were significantly predicted by students’ cumulative GPA, proving that both test formats could discriminate against students’ performances. The students in the higher GPA were expected to obtain a higher score on the blended test format and on the traditional test format. In particular, a sharp increase in the adjusted R square suggested the magnitude of improved prediction for the low-achieving group (*Reg 6*) in terms of blended assessment. The GPA was found to be a single significant predictor of each test score, but gender, age, and admission type were not significant in most of the regression models.

**Table 2:** Coefficient comparison for multiple regressions of cumulative GPA on student body and admission on 2019 CB and 2021 blended test scores

	2019 CB test scores			2021 Blended test scores		
	*REG 1* Total Students	*REG 2* High Achievers	*REG 3* Low Achievers	*REG 4* Total Students	*REG 5* High Achievers	*REG 6* Low Achievers
Cumulative score						
GPA	0.737** (9.606)	0.591** (4.673)	0.447** (2.899)	0.477** (4.954)	0.457** (3.296)	0.395** (2.998)
Gender	0.046 (0.595)	0.180 (1.360)	− 0.020 (− 0.130)	− 0.105 (− 1.036)	0.260 (1.827)	− 0.470** (− 3.484)
Student body						
Age	0.016 (0.149)	0.128 (0.653)	− 0.017 (− 0.077)	0.054 (0.416)	0.269 (1.340)	− 0.012 (− 0.074)
Admission						
Admission type	− 0.021 (− 0.198)	− 0.016 (− 0.080)	− 0.047 (− 0.232)	0.050 (0.382)	− 0.209 (− 1.050)	0.143 (0.867)
F statistic (*p*-value)	26.1 (< 0.001)	6.079 (< 0.001)	2.522 (0.057)	6.44 (< 0.001)	3.34 (< 0.001)	6.37 (< 0.001)
R square	0.554	0.372	0.210	0.235	0.246	0.415
Adjusted R squared	0.539	0.311	0.127	0.198	0.172	0.353

^*^p < .05 ** p < *.01* (*t* values in parentheses) CB test indicates traditional closed-book tests, and blended tests includes in-person closed-book and online open-book tests. GPA means a cumulative grade point average of individual students. Students are admitted to either a 4 + 4 dental program, comprising an undergraduate degree and Doctor of Dental Surgery degree (DDS) or a 3 + 4 program, which is a single program combining a BS and a DDS

### Perception survey on a blended assessment

An analysis of the responses to the open-ended questions exhibited several highly topical themes that frequently recurred (Table 3). In the theme of burdens of exams, the most frequently mentioned advantage of OBEs was reduced burden on the students about the test. Most of the students reported that they were able to reduce unnecessary memorization for the exam and remember important information, thus reducing the strain of preparing for it.

‘I always felt that I had to memorize for the test rather than what I had to know while studying, and it was good to be able to remember the significant points.’

‘The test was open-book, so the burden of memorizing unnecessary details was reduced. However, there seemed to be no significant difference in study time in that logical thinking was required.’

**Table 3:** Topical themes of dental students’ self-perceived responses about blended assessment (*N* = 89)

Category	Sub-categories	N	%
1. Exam Burden	1–1. [online OBEs] Reduced burden of memorizing unnecessary details; relieved anxiety	21	24
	1–2. No significant difference in study time and mental efforts between CBEs and OBEs	17	19
2. Learning Effects of Exams	2–1. [online OBEs] Prompting an evaluation of all knowledge of clinical practices; employing logical thinking processes; enabling students to understand study materials comprehensively; encouraging deep learning	34	38
	2–2. [class CBEs] Motivating students to spend more time gaining domain knowledge; knowledge retained for a longer period	17	19
3. Exam Environment	3–1. [online OBEs] Bringing about fairness issues; invigilators should be required	8	9
	3–2. [online OBEs] Guaranteeing individual safety during the pandemic	7	8

Percentages indicate how many responses are assigned into each category, and multiple answers were allowed

In the theme categorized as learning effects of exams, the students mentioned that CBEs motivated them to spend more time gaining domain knowledge and they could spend more time trying to understand study materials comprehensively. In particular, the students’ self-perceived responses denoted that OBEs can be applied to actual situations and they are instrumental in developing critical thinking skills.

‘It seems that more than 90% of the written tests at dental schools are traditionally evaluated based on knowledge memorization, but OBEs were very meaningful to evaluate all the knowledge I learned during the semester in relation to clinical practices.’

‘Based on what we learned in class, I think the critical thinking ability to determine what treatment to do based on the knowledge we have when we encounter a patient in real-life clinical practice is also a very important factor for us to have. In this test, it was not just memorization, but it was good to learn and describe the process of working out the treatment process ourselves.’

On the category of environments of exams, the issue of fairness was one of the main problems the students pointed out since the final open-book test was implemented online.

‘There may still exist the possibility of sharing answers, so it is unfair in terms of equity. It would be fairer to take an open-book exam together.’

‘The OB test is so good, but I don't think it can be overlooked that online OBEs cannot be free from the issue of fairness in evaluation.’

## Discussions

This study attempted to examine whether it is effective to use a blended assessment of CBEs and OBEs to reduce test anxiety and enhance academic performance among dental students. This study aimed to determine whether a blended assessment can discriminately measure the learning outcomes of dental students by comparing their academic achievement in this form assessment with that of traditional assessment.

The findings revealed that the anxiety level of the OBEs did not decrease compared to that of the CBEs. In previous studies, OBEs have generally exhibited lower test anxiety [5], but in this study, anxiety about OBEs was found to be slightly higher than that of CBEs. This seems to suggest that the students felt no less anxious in the OBEs than in the CBEs and they experienced the same level of tension during the OBEs. In fact, most dental professors may be concerned that there are instances where OBEs tend to lessen students’ tension, thus negatively affecting exam preparation as compared to CBEs. However, students’ perceived responses indicated that OBEs can serve as a meaningful evaluation that maintains appropriate tension and that they spent as much time preparing for the OBEs as for the CBEs.

On the other hand, it has been reported that OBEs do not reduce test anxiety relative to CBEs among students who find OBEs unfamiliar and unpredictable and felt pressured to write answers using their knowledge [16, 17]. Some students’ responses indicated that the preparation for the OBEs was a burden, mainly due to a lack of understanding of how to prepare. Thus, informing students in advance that an exam will be open book and asking them to analyze clinical scenarios or perform problem-solving can change their approach to studying for the exam. In addition, medical educators should provide OB test samples to help students prepare for the OBEs before they encounter them in high-stakes settings to reduce anxious and target their preparation to the novel test format [15]. Assessment workshops for faculty training could be adopted to share pilot results and practical tips in adopting OBEs for their courses.

Test anxiety has reportedly been associated with poor performance in exams due to worrying about the outcome and experiencing negative emotions during the test [17]. Previous studies found a significant negative correlation between test anxiety and academic achievement, including students’ GPA [18]. Interestingly, a systematic review indicates that there was compelling evidence of substantial prevalence of anxiety disorders, particularly in women and young adults, which negatively influenced their accumulative academic performances [19]. In our sample, test anxiety level depending on test formats did not significantly differ with gender; however, the mean of test anxiety score (M = 2.22) among female students was slightly higher than that (M = 2.05) among male ones. It may be inferred that more pressure is placed on females than males to succeed in school, and academic counseling for female students should focus more on dealing with test anxiety [19].

In previous studies, the mean scores were similar for both OBEs and CBEs [20, 21]. However, in this study, on average, the final OBE scores for 2021 were lower than the final CBE scores in 2019. This suggests that this type of exams was unfamiliar to many learners. In this study, the students reported difficulty in preparing for the OBEs, as it was their first time sitting for this kind of test. In line with the findings of previous studies, high-achieving students exhibited superior performance in their cumulative GPA in both the traditional and blended assessments conducted in 2019 and 2021 [22]. Interestingly, an increased adjusted R square of the regression model found that the scores of students at a lower rank were better predicted by obtaining lower scores in a blended test format, which suggests that blended assessments would be more effective in monitoring the low-achieving group than traditional assessments. Thus, implementing a blended assessment could play a significant role in targeting the low-performing group and contributing to developing dental competencies, such as critical thinking ability, that are difficult to acquire through rote memorization alone.

For the OBEs to be discriminatory and meaningful, it is essential to design exams that require high contextual and high-order thinking. Notably, OBEs are basically designed not to evaluate learners’ knowledge but to evaluate their ability to solve real problems [22, 23]. Consistent with Bloom’s theory of taxonomy, the students’ self-perceived responses to the OBEs showed that they were motivated to primarily focus on understanding and synthesizing knowledge acquired in classes [24]. Previous studies reported that there was no significant difference between evaluations of lower-order thinking skills in open- and closed-book assessment, but OBEs had a greater effect on developing higher-order thinking ability, such as application, analyzing, evaluating, and creating [8]. While a knowledge-based class that tests learners based on rote memorization makes it easy to reach lower-order learning goals, an open-book assessment encourages learners to reach higher-order learning goals by synthesizing knowledge and promoting long-term memory.

Although the need to establish an online evaluation platform for dental education has been recognized, the amount of discussion and research on the topic has been inadequate [25, 26]. Dental education is confronting new challenges worldwide due to the COVID-19 pandemic, and most lectures have been switched from offline to online learning platforms to maintain social distancing [27, 28]. The context of the COVID-19 pandemic gives rise to the question of assessment methods in education in the healthcare profession [29]. In the survey, the students indicated that taking an OBE in a comfortable situation made them feel relieved and less stressed, as well as guaranteeing their individual safety from COVID-19. Because online evaluation must be done where the instructor and the learner are not in the same space, online open-book assessment can be an alternative to traditional assessment under a situation that does not permit face-to-face interactions.

The perception survey also showed that the OBEs were more effective in developing dental competencies than traditional CBEs in terms of reducing the learning burden and enhancing the learning effects. Generally, the type of exam adopted can influence how the exam is studied for. It has been reported that students preparing for OBEs tend to pay more attention and integrate their knowledge during class compared to those preparing for the traditional CBEs [6, 30]. In an open-ended survey on the assessment, the students perceived that OBEs reduced the burden of memorizing unnecessary details; therefore, they could spend more time concentrating on problem-solving than on rote memorization. Thus, it may be inferred that employing the OB test format provides students with greater opportunity to be critical and develop the higher-order thinking skills required of dental graduates.

Improvements were also suggested in the blended test format. In online OBEs, fairness was cited as the first issue of concern. While the midterm CBEs were conducted through in-person attendance and supervised by instructors, the final OBEs were implemented online without physical attendance. For the online OB assessment, Turnitin, a text matching system within an electronic document, was used to check whether the students’ work matched with previously submitted works. In addition, the OB answers were evaluated using clear standards that employed a rubric and through a dual scoring process that involved two experienced instructors. Recent research findings have supported that experienced markers can differentiate between genuine works and those completed by third parties [31]. However, as per previous studies and the students’ responses, there is still issue of fairness needs to be carefully addressed in blended assessments [20, 31].

This study is not free from limitations. Caution is required when translating this study’s findings to other medical and dental course settings. Because the sampling is a census, it was almost impossible to control all factors that threaten the validity of this nonrandomized, quasi-experimental study. The study population consisted of two different cohorts, which took the same course under the same instruction. We assumed that the cohort populations did not significantly differ in terms of gender, age, and admission requirements. However, the pandemic occurred in 2020, which inevitably characterized cohort 2019 as pre-pandemic and cohort 2021 as post-pandemic. Naturally occurring changes in study design over time may be confused with intervention effects. Accordingly, before applying the findings to different contexts in health profession education, convincing evidence for causal links needs to be examined so that instructors weigh the trade-offs between close- and open-book exams and verify whether there may be a factor other than test formats that affects student performance. To evaluate the effectiveness of this blended approach, it is imperative to use it repeatedly in different disciplines and classes.

In addition, the 2019 traditional assessment used question types such as true or false, multiple-choice, and short-answer questions that are associated with rote learning, but the 2021 OB questions were designed to assess the students’ problem-solving skills. Finally, the validity of the OB assessment methods used in this study need to be tested by proving whether they really worked as authentic, real-life-like assessments to improve students’ higher-order thinking [29]. In follow-up assessment studies, the roles and effects of invigilation in the online exams should be carefully monitored, and the extent to which OBEs allow students to access resources, including self-made materials, needs to be examined in terms of exam types.

## Conclusion

This study investigated the effects of a blended assessment of CBEs and OBEs on anxiety and performance in dental students. A blended assessment was as effective as traditional assessment in distinguishing good from poor students. No significant differences were found in dental students’ anxiety level between traditional and blended assessments. Both types indicated a similar trend of lower scores in midterm exams followed by higher scores in final exams, but the lower-achieving group was better monitored by a blended assessment. In particular, an analysis of students’ self-perceived responses denoted what remains to be done for more effective assessment in terms of learning burden, learning effects, and fairness. This study also confirmed the feasibility of blended assessment that can be implemented in online and in-person educational environments. Moreover, it can be used as the groundwork to develop new models of assessment in dental education.

## Data Availability

The datasets used and/or analyzed during the present study are not publicly available due to limitations of ethical approval involving student data and anonymity but are available from the corresponding author on reasonable request.

## Abbreviations

CB: Closed-book
OB: Open book
CBEs: Closed-book examinations
OBEs: Open-book examinations
COVID: Coronavirus disease
